# Voltage dependence of the cannabinoid CB1 receptor

**DOI:** 10.3389/fphar.2022.1022275

**Published:** 2022-10-11

**Authors:** Esty Goldberger, Merav Tauber, Yair Ben-Chaim

**Affiliations:** Department of Natural and Life Sciences, The Open University of Israel, Ra’anana, Israel

**Keywords:** G protein couple receptors, *Xenopas* oocytes, voltage dependence, cannabinoid (CB) receptor 1, cannabinoids

## Abstract

Cannabinoids produce their characteristic effects mainly by binding to two types of G-protein coupled receptors (GPCRs), the CB1 and CB2 cannabinoid receptors. The CB1 receptor is the main cannabinoid receptor in the central nervous system, and it participates in many brain functions. Recent studies showed that membrane potential may serve as a novel modulatory modality of many GPCRs. Here, we used *Xenopus* oocytes as an expression system to examine whether membrane potential modulates the activity of the CB1 receptor. We found that the potencies of the endocannabinoid 2-AG and the phytocannabinoid THC in activating the receptor are voltage dependent; depolarization enhanced the potency of these agonists and decreased their dissociation from the receptor. This voltage dependence appears to be agonist dependent as the potency of the endocannabinoid anandamide (AEA) was voltage independent. The finding of this agonist-specific modulatory factor for the CB1 receptor may contribute to our future understanding of various physiological functions mediated by the endocannabinoid system.

## Introduction

G-protein coupled receptors (GPCRs) represent the largest membrane protein family and are of great physiological importance. In recent years, a new factor starts to emerge as a modulator of GPCRs activity. Several studies have shown, employing different approaches, that not only can GPCRs modulate membrane potential by activating ion channels, but that the membrane potential can modulate the affinity and activity of GPCRs. To date, many GPCRs including cholinergic (Ben-Chaim et al., 2003; Ben-Chaim et al., 2006; Navarro-Polanco et al., 2011), glutamatergic (Ohana et al., 2006), dopaminergic (Sahlholm et al., 2008a; Sahlholm et al., 2008c; Ågren and Sahlholm, 2020), adrenergic (Rinne et al., 2013) and purinergic receptors (Martinez-Pinna et al., 2004; Martinez-Pinna et al., 2005) have been shown to be voltage dependent.

The most studied voltage sensitive GPCRs are the muscarinic M1 and M2 receptors. For these receptors we have shown that their binding affinity is voltage sensitive; depolarization shifts the receptor into a low affinity state in the case of the M2R, and into a high affinity state in the case of the M1R (Ben-Chaim et al., 2003). Furthermore, it was found that depolarization induces movement of charges within these receptors that are correlated with the fraction of receptors that undergo a change in binding affinity (Ben-Chaim et al., 2006) indicating that the charge movement is indeed related to the voltage dependency of the affinity of the receptor. This conclusion was followed by a series of experiments that were aimed to investigate the molecular mechanism by which this receptor senses changes in voltage and translates them to changes in affinity (Dekel et al., 2012). Recently, we have identified a voltage sensing motif in the M2R and suggested that this “voltage sensor” is responsible for the voltage dependence of the M2R (Barchad-Avitzur et al., 2018).

Here, we aimed to extend our research to a new family of GPCRs, the cannabinoid receptors. Cannabinoids produce their characteristic behavioral effects as a consequence of binding to two types of GPCRs, the CB1 and CB2 cannabinoid receptors (Matsuda et al., 1990; Munro et al., 1993). The cannabinoid receptor 1 (CB1) is the most abundantly expressed GPCR in the brain (Marsicano and Lutz, 1999) and it is a therapeutically useful target involved in a wide variety of physiological processes such as: metabolic regulation, craving, pain, and anxiety (Mackie, 2006; Pacher et al., 2006; An et al., 2020)). The CB1 receptor is also involved in controlling the resting potential of excitable cells by activating and modulating several classes of ion channels (Turu and Hunyady, 2010). The role of the CB1 receptor in long term potentiation (LTP) and other forms of activity-dependent plasticity in the brain is now quite established (Carlson et al., 2002; Augustin and Lovinger, 2018; Winters and Vaughan, 2021; Augustin and Lovinger, 2022). Interestingly, some of these processes are known to be mediated by changes in membrane potential, e.g., depolarization-induced suppression of excitation and inhibition (Kreitzer and Regehr, 2001; Straiker and Mackie, 2005).

For several GPCRs, it has been proposed that voltage dependence is also agonist specific (Sahlholm et al., 2008b; Navarro-Polanco et al., 2011; Rinne et al., 2015; Ruland et al., 2020); namely, depolarization, that reduces the binding of one agonist to a given receptor, will have no effect, or will even have an opposite effect, on the binding of other agonists. This agonist specificity may have important implications both for the understanding of the mechanism that underlies voltage dependence of GPCRs and for the possible application of this knowledge in pharmaceutical design. In the field of cannabinoid receptors though, agonist specificity of the voltage dependence becomes a more pivotal question. This is because these receptors, unlike other GPCRs, have more than one endogenous ligand (endocannabinoids) (di Marzo and de Petrocellis, 2012). Thus, the particular voltage-dependencies of different endocannabinoids may serve as a novel regulatory modality of the signal transduction of these receptors by different agonists. Here, we show that the voltage dependence of the CB1 is agonist-specific.

## Materials and methods

### Ethics statement

All experimental procedures used in this study were performed in accordance with relevant guidelines and regulations and were approved by the Hebrew University’s Animal Care and Use Committee (Ethical approval number NS-11-12909-3).

### Preparation of cRNA and oocytes

cDNA plasmids of the two subunits of the GIRK (GIRK1 and GIRK2), the CB1 receptor (kindly provided by Ken Mackie, Indiana University, Bloomington, Indiana) and the α subunit of the G-protein (Gαi3) were linearized with the appropriate restriction enzymes (Friedman et al., 2020). The linearized plasmids were transcribed *in vitro* using a standard procedure.

*Xenopus laevis* oocytes were isolated and incubated in NDE96 solution composed of ND96 (in mM: 96 NaCl, 2 KCl, 1 CaCl2, 1 MgCl2, 5 Hepes, with pH adjusted to 7.5 with NaOH) with the addition of 2.5 mM Na+ pyruvate, 100 units/ml penicillin, and 100 μg/ml streptomycin (Tauber and Ben Chaim, 2022). A day after their isolation, the oocytes were injected with cRNAs of CB1 receptor (2 ng) and GIRK1 and GIRK2 (200 pg each). In addition, cRNA of Gαi3 (1,000 pg) was injected to decrease the basal GIRK current (I_K_) and to improve the relative activation by the agonist (Peleg et al., 2002). Injection of Gαi3 proved to decrease I_K_ by about 3-fold.

Chemicals were purchased from Sigma Israel (Rehovot, Israel). THC was kindly provided by Bazelet group (Or Akiva, Israel).

### Current measurements

The currents were measured 4–7 days after cRNA injection and were recorded using the standard two-electrode voltage clamp technique (Axoclamp 2B amplifier, Axon Instruments, Foster City, CA). Each oocyte was placed in the recording bath containing ND96 solution and was impaled with two electrodes pulled from 1.5-mm Clark capillaries (CEI, Pangboure, England). Both electrodes were filled with 3M KCl solution and the electrodes resistances were 1-5 MΩ. The CB1-mediated GIRK currents were measured in a 24 mM K+ solution (in mM: 72 NaCl, 24 KCl, 1 CaCl2, 1 MgCl2, 5 Hepes, with pH adjusted to 7.5 with KOH) (Friedman et al., 2020). pCLAMP10 software (Axon Instruments) was used for data acquisition and analysis.

### Data analysis

**FIGURE 1:**
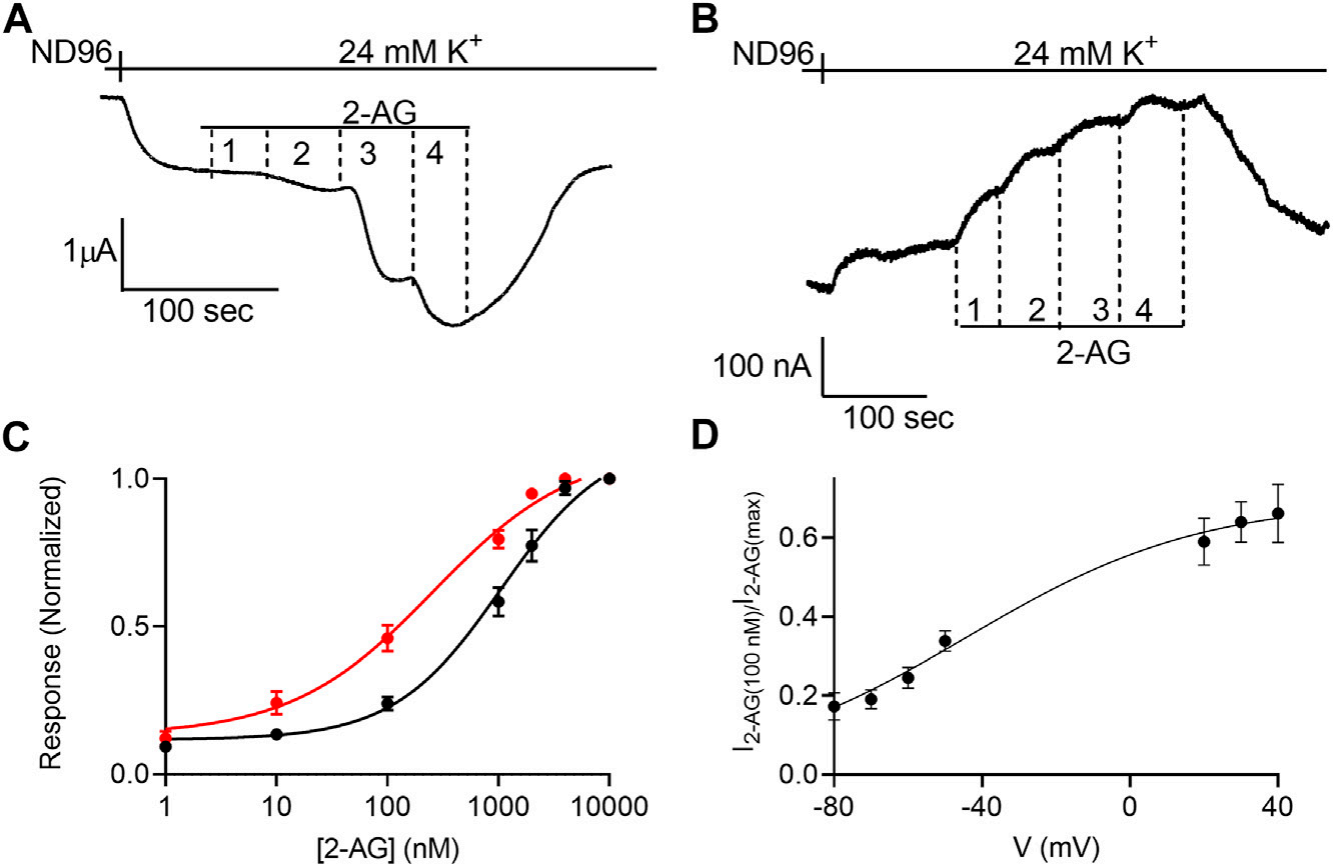
Voltage dependence of the potency of 2-AG. **(A)** and **(B)**. Measurement of the relationship between 2-AG concentration and GIRK currents at -80 mV and at +40 mV, respectively. Basal GIRK current is evolved following replacement of the solution to a high K+ solution. Then, 4 different 2-AG concentrations (100, 1,000, 2000 and 10000 nM) were applied and the response for each concentration was measured. **(C)**. Dose response curves for several 2-AG concentrations at −80 mV (black symbols and line; *n* = 19, 45, 51, 39, 9, 19 and 39 for 1, 10, 100, 1,000, 2000, 4,000 nM respectively) and at +40 mV (red symbols and line; *n* = 8, 26, 28, 15, 19, 12 and 39 for 1, 10, 100, 1,000, 2000, 4,000 nM respectively). The responses are normalized to the response evoked by 10000 nM 2-AG at each holding potential. **(D)** The dependence of the relative activation of the receptor by 100 nM 2-AG in voltage. Responses are normalized to the response evoked by 10000 nM 2-AG at each holding potential.

**FIGURE 2:**
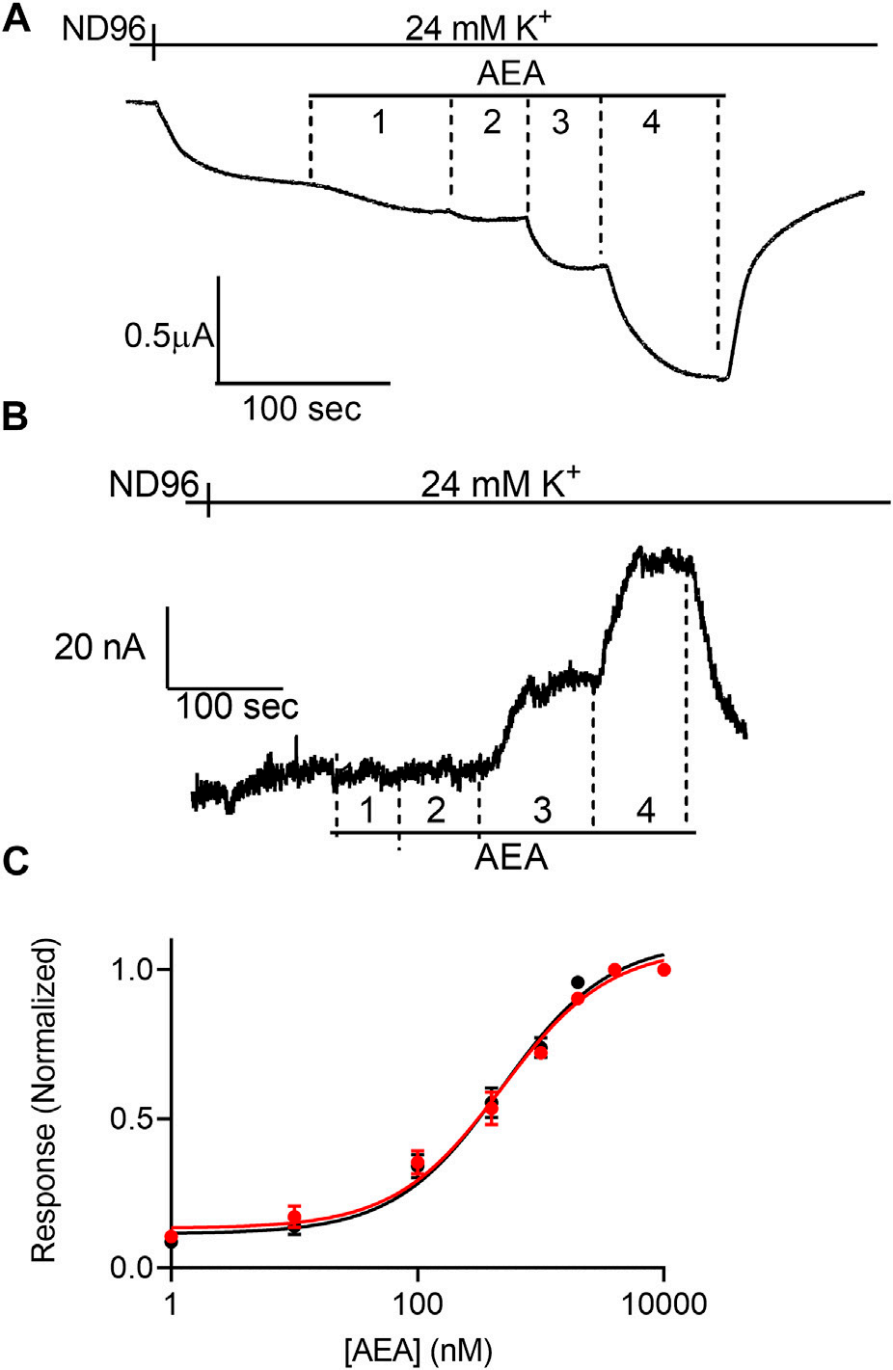
Voltage dependence of the potency of AEA. **(A)** and **(B)**. Measurement of the relationship between AEA concentration and GIRK currents at -80 mV and at +40 mV, respectively. Basal GIRK current is evolved following replacement of the solution to a high K+ solution. Then, 4 different AEA concentrations (1, 10, 400 and 10000 nM) were applied and the response for each concentration was measured. **(C)**. Dose response curves for several AEA concentrations at −80 mV (black symbols and line; *n* = 31, 20, 20, 14, 25, 11, 8 and 41 for 1, 10, 100, 400, 1,000, 2000, 4,000, and 10000 nM, respectively) and at +40 mV (red symbols and line; n = 8, 10, 9, 8, 17, 11, 8 and 30 for 1, 10, 100, 400, 1,000, 2000, 4,000, and 10000 nM, respectively). The responses are normalized to the response evoked by 10000 nM AEA at each holding potential.

The dose response curves (Figures 1C, 2C, 5A) were fitted by Eq. 1:

$$Y=\text{Bottom}+\left(X^{\text{Hill}\;\text{slope}}\right)*\left(\text{Top}-\text{Bottom}\right)/\left(X^{\text{Hill}\;\text{Slope}}{+\text{EC}50}^{\text{HillSlope}}\right)$$ (1)

where Y is the normalized response, X is the concentration of agonist, Hill slope is the slope factor and EC_50_ is the agonist concentration that gives the half-maximal response.

The dependence of agonist potency on membrane potential (Figures 1D, 5D) was analyzed by fitting Boltzmann equation (Eq. 2) to the data.

$$Y=\text{Bottom}+\left(\text{Top}-\text{Bottom}\right)/\left(1+e\left({\text{ze}}^{\left(V50-X\right)/\text{kT}}\right)\right)$$ (2)

where Y is the normalized response, X is the membrane potential, z is the slope, e the elementary electric charge*, k* is the Boltzmann constant and T is the temperature.

The time constant of the decay of CB1 receptor-activated GIRK current was extracted by fitting a single exponential to the decay of the current. We began the fit after the current declined to 80% of its maximal level (Ben Chaim et al., 2013).

### Statistical evaluation

Statistical analysis was conducted using Prism GraphPad software. Significance was evaluated by Student’s two-tailed *t*-test. Estimating the difference between the EC_50_ values was conducted by the extra-sum-of-squares F test.

## Results

### Membrane potential affects the dependence of CB1 receptor-mediated GIRK response on 2-AG concentration

To study the voltage dependence of the potency of 2-AG to activate the CB1, *Xenopus* oocytes were injected with cRNAs of proteins involved in the pathway leading to activation of K^+^ currents by the CB1 receptor *via* βγ subunits of the G-proteins: CB1 receptor, the subunits of the GIRK channel (GIRK1 and GIRK2), and the Gαi3 subunit.

We first verified that the agonists that will be used in this study do not exert a receptor-independent effect on the GIRK channels. To do so we measured the effect of 2-Arachidonoylglycerol (2-AG), anandamide (AEA) and (-)-Δ9-Tetrahydrocannabinol (THC) on oocytes expressing the GIRK channel but not the CB1 receptor (Supplementary Figures S1–S3, respectively). The results show that the three agonists do not have any direct effect on the GIRK channel at any membrane potential.

Next, the dependence of the 2-AG-induced K+ current (I_2-AG_) on 2-AG concentration (dose-response, DR) was measured at two holding potentials: –80 mV and +40 mV. Figures 1A,B depicts the basic experimental protocol for four 2-AG concentration. The oocyte was voltage-clamped to either –80 mV (Figure 1A) or +40 mV (Figure 1B), in a low K+ (2 mM K+) solution, ND96. Basal GIRK current (I_K_) was developed upon replacement of the ND96 by the 24 mM K+ solution. Then, four concentrations of 2-AG were applied sequentially, giving rise to an additional GIRK current, denoted I_2-AG_. I_2-AG_ was terminated upon washout of 2-AG. Employing this basic experimental protocol, full DR curves at the two holding potentials were constructed. To compensate for the intrinsically different GIRK currents obtained at the two holding potentials of −80 mV and +40 mV in a single oocyte, and to be able to compare between oocytes, for each holding potential, I_2-AG_ at any particular 2-AG concentration was normalized to I_2-AG_ obtained at a saturating concentration of 2-AG at the same holding potential. Figure 1C depicts the cumulative results from 10 experiments. Because we were not able to record at both holding potentials in all oocytes, the results are both from oocytes where data was obtained from one of the holding potentials and recordings where the same oocyte was subjected to both holding potentials. The results of the latter were not different from the cumulative results. It is seen that membrane potential affects the apparent affinity of 2-AG toward the CB1 receptor. Specifically, depolarization enhances the potency of this ligand in activating the CB1 receptor. The EC_50_ was 1,060 nM at −80 mV and 259 nM at +40 mV (the two EC_50_ values are significantly different extra-sum-of-squares F test; *p* = 0.022). To further evaluate the voltage dependence of the potency of 2-AG toward the CB1 receptor we measured the activation of the receptor by 2-AG at several holding potentials. To this end, we measured GIRK currents at various holding potentials ranging from -80 mV to +40 mV at 10 mV increments before application of 2-AG and following application of 2 concentrations of 2-AG, 100 nM and 10 μM; Supplementary Figure S4). From such measurements, the relative activation of the CB1 receptor by 100 nM 2-AG was evaluated. Figure 1D shows the potency of 2-AG between −80 mV and +40 mV. Currents at voltages between −40 mV and +10 mV were too small to be reliably analyzed due to the low driving force of the GIRK channel at this voltage range. Fitting Boltzmann equation (Eq. 2) to the data revealed that voltage is effective in physiological membrane potentials, with V_50_ (the voltage that shows half-maximal effect) of −45 mV and a slope of the curve, z, of 0.8 eV.

### The potency of anandamide toward the CB1 receptor is voltage-insensitive

Next, we examined the effect of membrane potential on the apparent affinity of anandamide (AEA), a second endocannabinoid. To this end, the experiments described above were repeated with AEA as the agonist. Figures 2A,B show representative recordings of the response of CB1 receptor expressing oocytes to application of 4 concentrations of AEA at −80 mV and +40 mV, respectively. From 15 such experiments full DR curves were constructed at the two holding potentials (Figure 2C). It is seen, that in contrast to the voltage dependence of the apparent affinity of 2-AG, the activation of the CB1 receptor by AEA was voltage-independent. The EC_50_ was 449 nM at -80 mV and 554 nM at +40 mV (the two EC_50_ values are not significantly different, extra-sum-of-squares F test; *p* = 0.3).

### Voltage affects the dissociation rate of 2-AG from the CB1 receptor

**FIGURE 3:**
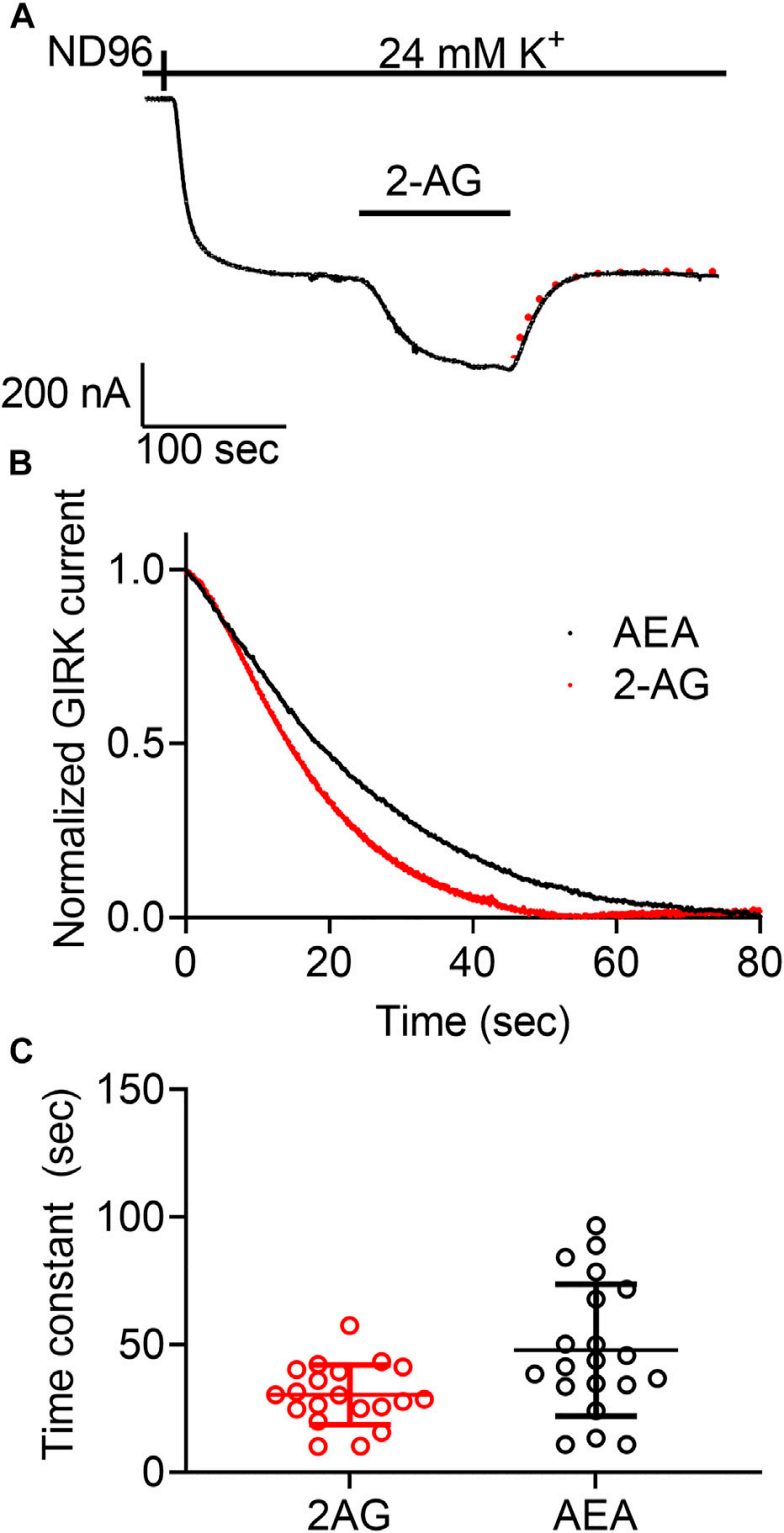
Measurement of the dissociation of agonist from the CB1 receptor by measuring the deactivation of CB1 receptor-evoked GIRK current following agonist washout. **(A)** An example from one oocyte. Current evoked by 2-AG was deactivated by washout of the2-AG. The dashed red line represents an exponential fit to the decay of the current. **(B)** A comparison of the decay of GIRK currents evoked by AEA (black) or 2-AG (red) following washout of the agonist at time zero. The currents were normalized to enable comparison between the two recordings. **(C)** Results from 20 oocytes with each agonist. The time constant of the decay of AEA-evoked currents is significantly higher than that of 2-AG evoked currents.

For the M2R we confirmed the voltage sensitivity by measuring the voltage dependence of the dissociation of the agonist from the receptor. We have shown that depolarization exerts its effect on the apparent affinity of the receptor by shifting the receptor between two states that differ in their dissociation rate constants (Ben Chaim et al., 2013). Similar voltage-dependence was also observed for the dissociation of glutamate from the metabotropic glutamate type 3 receptor (Ohana et al., 2006). We thus examined whether voltage has a similar effect on the CB1 receptor. To do so, we evaluated the dissociation rate constant of an agonist from the receptor by measuring the deactivation time of the CB1 receptor-activated GIRK currents. To this end, the following experiment was conducted (Figure 3A): Oocytes expressing the CB1 receptor and the GIRK channel were voltage clamped to -80 mV. An agonist is then applied, activating CB1 receptor-induced GIRK currents. After the current reaches a plateau, the agonist is washed out and the CB1 receptor-induced GIRK current declines. In the M2R and the mGluR3, it has been shown that the decline of these currents following the washout of the agonist reflects the dissociation rate constant of the agonist from the receptor (Ohana et al., 2006; Ben Chaim et al., 2013). To determine whether this is also the case for CB1 receptor-activated GIRK currents, we compared the dissociation rate constants of GIRK currents following the washout of the two agonists, AEA and 2-AG. As these endocannabinoids exhibit different affinities toward the CB1 receptor (see Figures 1C, 2C), if the decline of the GIRK currents does indeed reflect the dissociation of the agonist from the receptor, it is expected that the measured decline will be different for the two agonists. If, on the other hand, the decline of the currents is dictated by some other downstream process(s), then the measurements are not expected to be contingent on the agonist used. Figure 3B depicts a comparison of the decay of the current evoked by the two agonists. To avoid any variation that may be due to different experimental conditions, this comparison was done for recordings conducted from the same oocytes. From such recordings, the time constant of the decay was extracted by fitting a standard exponential equation to the decay. In order to avoid a possible contribution of re-association of the agonist to the receptor we started the fit only after the current decayed to 80% from its maximal amplitude. At this time no residual agonist is expected to be present at the recording chamber. The collected results (Figure 3C, *n* = 20) show that the dissociation time constant of AEA from the receptor (mean±SD = 47.8 ± 25.8 s) is significantly slower than that of 2-AG (30.4 ± 11.7 s). The difference observed between the decay times of the two agonists (see collected results in Figure 3C) suggests that the decline rate of the GIRK current does, in fact, depend on the agonist. Therefore, this parameter may be used as a measure for agonist dissociation rate.

**FIGURE 4:**
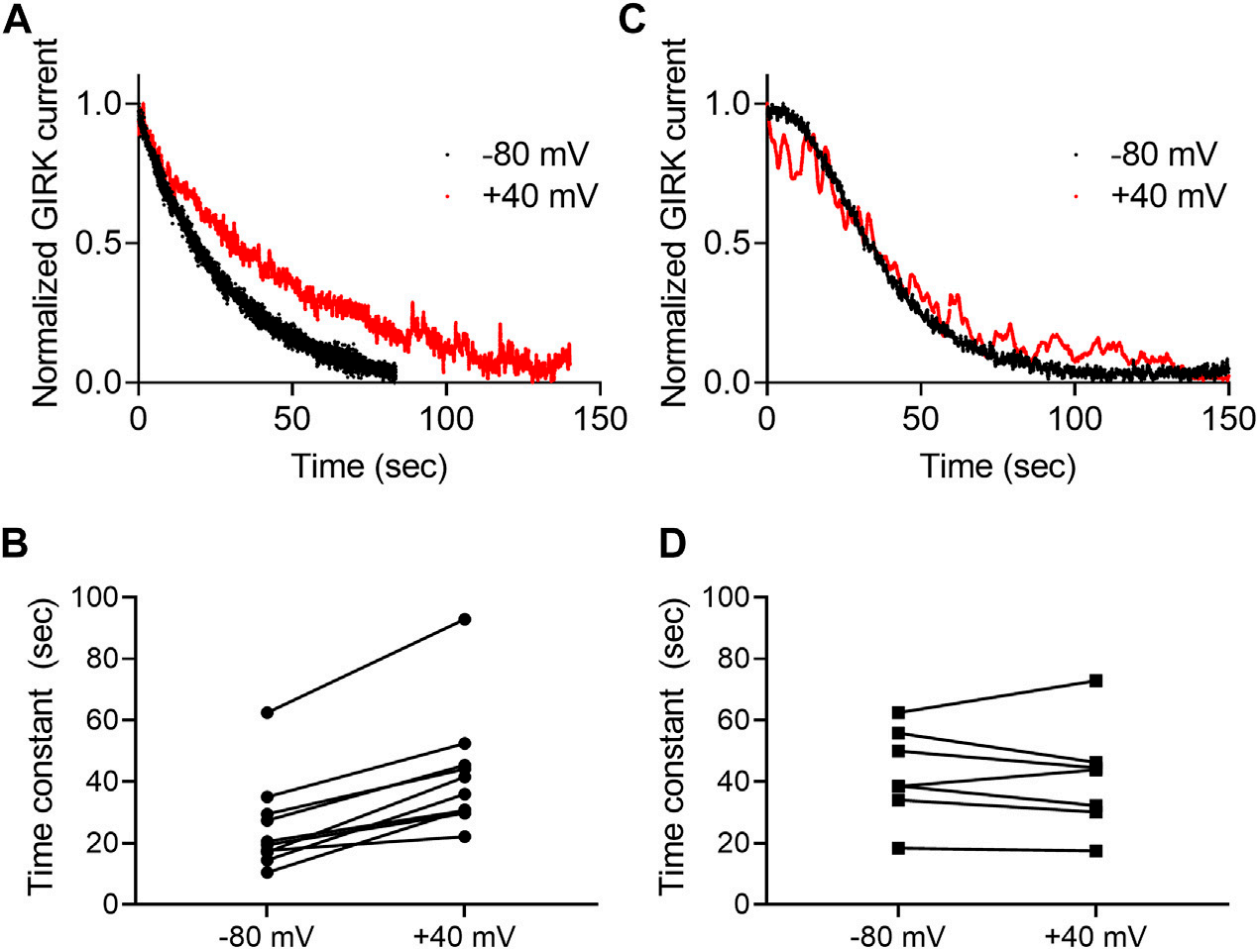
Voltage dependence of the dissociation of agonist from the CB1 receptor. **(A)** And **(C)**A comparison of the decay of GIRK currents at −80 mV (black) or at +40 mV (red). The currents were evoked following activation with 2-AG **(A)** or AEA **(C)**. The washout of the agonists took place at time 0 and the currents were normalized to enable comparison between the two recordings. **(B)** and **(D)**. Time constants of decay measured of −80 mV and +40 mV. The currents were evoked following activation with 2-AG **(B)** or AEA **(D)**.Each two data points connected with a line represent the same oocyte at the two holding potentials.

Next, we asked whether the dissociation rate constant is voltage dependent. To do so, the experiment described in Figure 3A was repeated at two holding potentials, −80 mV and +40 mV for each oocyte. Examples of such recordings using 2-AG and AEA are depicted in Figures 4A,B, respectively. Figures 4C,D show the collected results from 10 oocytes activated by 2-AG and seven oocytes activated by AEA. In each case, the time constant of the decay was measured at both membrane potentials at the same oocyte. It is seen that when the CB1 receptor was activated by 2-AG, the time constant of deactivation was voltage dependent; it was faster at resting potential than under depolarization (the mean time constant was 25.4 ± 14.9 at -80 mV and 42.6 ± 19.8 at +40 mV, *p* <0.0001, paired *t*-test). This suggests that the dissociation of 2-AG from the receptor is slower at +40 mV, consistent with higher affinity under depolarization. On the other hand, when AEA was the activating ligand, the decay of the GIRK current following washout occurred at similar rate at both membrane potentials (mean time constant was 42.5 ± 14.8 s at -80 mV and 41.1 ± 17.4 s at +40 mV, *p* = 0.6, paired *t*-test), again consistent with the observation that the affinity of AEA is not voltage dependent.

### The potency of THC toward the CB1 receptor is voltage-sensitive

**FIGURE 5:**
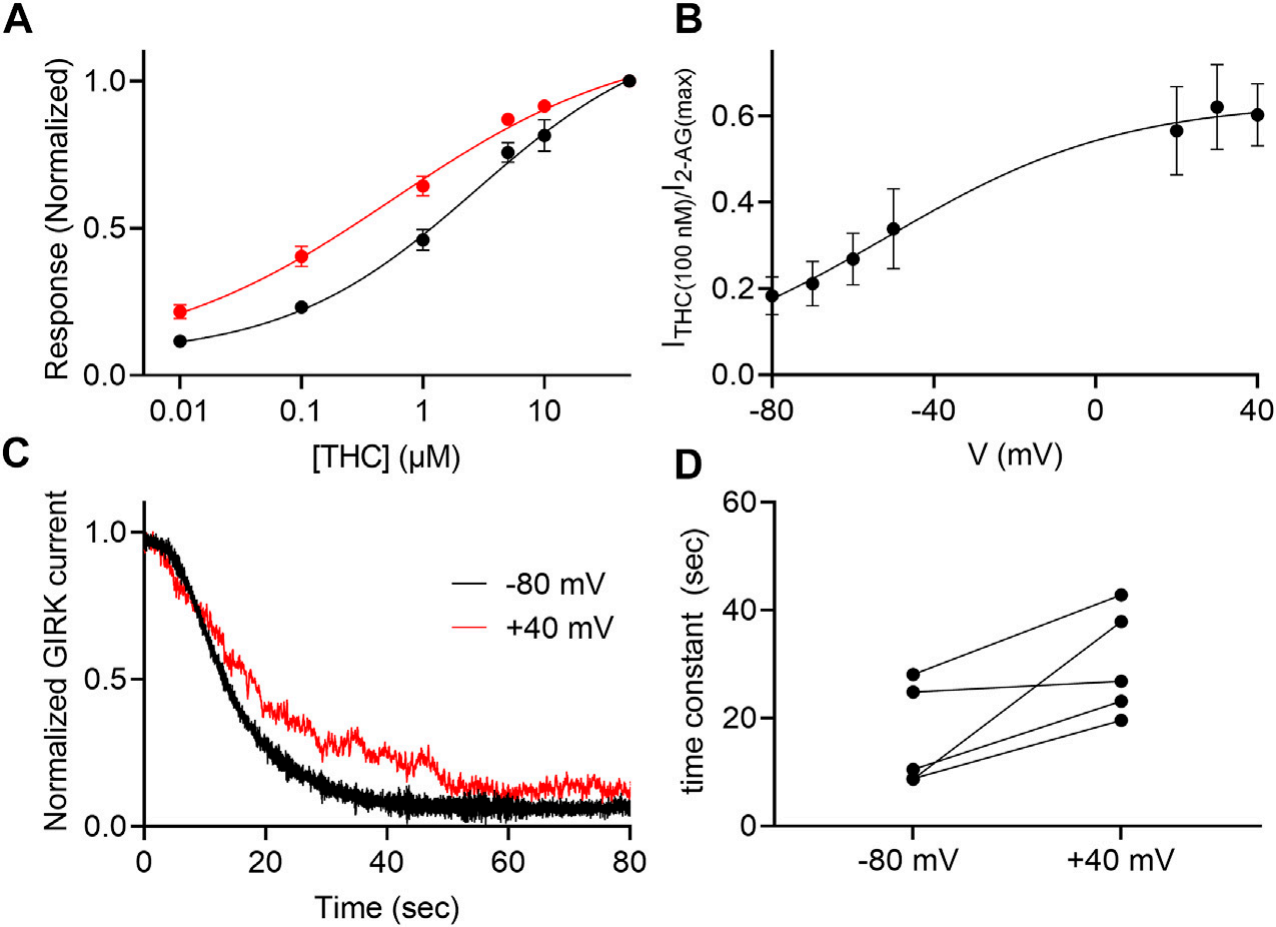
Voltage dependence of the potency of THC. **(A)** Dose response curves for several THC concentrations at −80 mV (black symbols and line; *n* = 29, 29, 29, 13, 12 and 30 for 1, 10, 100, 1,000, 5,000, 10000, and 50000 nM, respectively) and at +40 mV (red symbols and line; *n* = 18, 18, 18, 14, 8 and 21 for 1, 10, 100, 1,000, 5,000, 10000, and 50000 nM, respectively). The responses are normalized to the response evoked by 20 µM THC at each holding potential. **(B)** The dependence of the relative activation of the receptor by 100 nM THC in voltage. Responses are normalized to the response evoked by 50000 nM THC at each holding potential. **(C)** A comparison of the decay of GIRK currents at -80 mV (black) or at +40 mV (red). The washout of the agonists took place at time 0 and the currents were normalized to enable comparison between the two recordings. **(D)** Time constants of decay measured of −80 mV and +40 mV. Each two data points connected with a line represent the same oocyte at the two holding potentials.

THC is the major active psychotropic component of the marijuana plant, Cannabis sativa, and it is widely used both for recreational purposes and as a treatment for various conditions. We therefore examined the voltage dependence of the activation of the GIRK channel by this agonist. To this end we measured, as described above, the dose-response relationship of THC activated GIRK currents. Figure 5A depicts DR curves obtained at -80 mV and at +40 mV. It is seen that the potency of THC in activating the CB1 receptor is voltage dependent. The EC_50_ was 2,736 nM at −80 mV and 568 nM at +40 mV (the two EC_50_ values are significantly different, extra-sum-of-squares F test; *p* = 0.03). As for 2-AG, we analyzed the voltage dependence of the potency of the THC as well, repeating the experiment described in Figure 1D. From such measurements, the relative activation of the CB1 receptor by 100 nM THC was evaluated. Figure 5B shows the potency of THC between -80 mV and +40 mV. Fitting Eq. 2 to the data revealed similar V_50_ of −49 mV and similar slope of the curve (z = 0.87 eV), suggesting a similar voltage dependence of the CB1 receptor toward THC. We also tested whether the dissociation of THC from the receptor is voltage dependent. The results are shown in Figures 5C,D. As seen, the results are compatible with the conclusion that the dissociation of THC from the CB1 receptor is voltage dependent; the dissociation time constant is smaller at -80 mV (16.2 ± 9.5 s) than at +40 mV (30.1 ± 9.9 s; the difference was statistically significant, *p = 0.034*, paired *t*-test).

## Discussion

Voltage dependence is emerging as a general property of GPCRs. So far, voltage dependence has been demonstrated for various GPCRs, including receptors for acetylcholine, adrenaline, dopamine, and glutamate. Recently, the first example of voltage dependent lipid-activated GPCR has been demonstrated with the measurement of voltage dependent activation of prostanoid receptors (Kurz et al., 2020). Here, we show that the cannabinoid CB1 receptor is also voltage dependent. Namely, the endocannabinoid 2-AG has higher potency in activating the receptor under depolarization than at resting potential. Similar voltage dependence was observed for the potency of THC toward the receptor. This is reflected also by the slower dissociation of the 2-AG and THC from the receptor under depolarization. Depolarization enhanced the potency of 2-AG toward the CB1 receptor by about 4-fold. This is similar to the effect observed for the potency of acetylcholine toward the M2R (Friedman et al., 2020) and of glutamate toward the metabotropic glutamate 3 receptor (Ohana et al., 2006) and somewhat smaller than that observed for the D2 dopamine receptor (∼10 fold) (Sahlholm et al., 2008c) and the 5-HT1A receptor (∼20 fold) (Tauber and ben Chaim, 2022). All these studies utilized similar GIRK channel activation assay. Comparison to studies that used other assays may not be ad te. Comparing the voltage dependence of the CB1 receptor to that of other GPCRs reveals V_50_ values and a shallow slope that is similar to reported for other GPCRs (Ben-Chaim et al., 2006; Navarro-Polanco et al., 2011; Rinne et al., 2015). This observation may suggest that a similar molecular mechanism underlies the effect of voltage on this receptor.

The molecular mechanism that underlies the voltage dependence of GPCRs is not fully understood, and several mechanisms were suggested. Based on modeling approaches, a link between the modulatory effects of Na^+^ and the voltage dependence of GPCRs has been suggested, predicting movement of Na^+^ from its binding site upon changes in membrane potential (Vickery et al., 2016b; 2016a). However, recent experimental evidence showed that removing extracellular Na^+^ did not abolish the voltage dependence of GPCRs (Friedman et al., 2020; Tauber and ben Chaim, 2022). Furthermore, receptors where the principle Na^+^ binding residue (D2.50, Ballesteros–Weinstein numbering) were mutated retain their voltage dependence (Barchad-Avitzur et al., 2018; Ågren et al., 2018).

For the most studied voltage dependent GPCRs, the muscarinic receptors, it has been suggested that polar tyrosine residues in the vicinity of the binding pocket form a “tyrosine lid” that may serve as voltage sensing elements, and that their movement upon depolarization lead to the conformational change that underlies the functional change in affinity (Barchad-Avitzur et al., 2018). Cannabinoid receptors do not contain a similar “lid” structure. Thus, it is likely that their voltage sensing element differs from that previously described for the M2R. A possible candidate to play a role in the voltage dependence of the CB1 receptor is residue Lys192. This charged residue is located near the ligand binding site and was implicated in ligand binding (Chin et al., 2002).

It has been suggested that G protein coupling plays a role in determining the voltage dependence of GPCRs (Ben-Chaim et al., 2003, 2006, 2019). In line with this suggestion, a correlation seems to exist between the coupled G protein and the direction of the voltage dependence. Namely, in most cases Go/i and Gs coupled receptors such as the M2R, the D2R, and mGluR3 showed reduced potency under depolarization, while Gq coupled receptors, such as the M1R, the mGlur1, and the P2Y receptors showed enhanced potency under depolarization. The Gq coupled M3 muscarinic receptor is an exception as its potency is reduced by depolarization (Rinne et al., 2015). To date, the current study presents the first, to our knowledge, Go/i coupled receptor that exhibits enhanced potency of activation by its natural ligand, 2-AG, under depolarization. This observation may be in line with studies that showed that this receptor may also induce Gq activated-pathways in some cases (Lauckner et al., 2005). Furthermore, this observation may be consistent with the notion that the structural rearrangement during the activation of the CB1 receptor differs significantly from that of amine activated GPCRs (Hua et al., 2016, 2017). Such different structural changes are also reflected by the receptor-G protein interphase of this receptor which differs from that of other structurally resolved active GRPC-G protein complexes (Krishna Kumar et al., 2019).

For some receptors, it was found that their voltage dependence is agonist-specific. Specifically, depolarization may increase the potency of one ligand to activate the receptor, while it has the opposite effect, or no effect, on the potencies of other ligands (Sahlholm et al., 2008b, 2008a; Navarro-Polanco et al., 2011; Rinne et al., 2015; Moreno-Galindo et al., 2016; Ruland et al., 2020). This phenomenon may shed light on the molecular mechanism of the voltage dependence, and may even have pharmaceutical implications. However, because most voltage dependent GPCRs studied are physiologically activated by their single cognate endogenous agonist, such agonist specificity is not expected to have physiological meaning. Cannabinoid receptors, on the other hand, have at least two endogenous ligands (di Marzo and de Petrocellis, 2012). Therefore, the particular voltage-dependencies of different endocannabinoids may serve as a novel regulatory modality of the signal transduction of these receptors by different agonists at different tissues and physiological functions.

The physiological importance of voltage dependence of GPCRs has been demonstrated in several processes, including cardiac function (Moreno-Galindo et al., 2011, 2016), neurotransmitter release (Parnas and Parnas, 2010; Kupchik et al., 2011) and, recently, muscarinic receptor mediated synaptic plasticity and memory (Rozenfeld et al., 2021). The CB1 receptor is known to play a role in many synaptic plasticity mechanisms, some of them were shown to be depolarization induced (Bura et al., 2017; Augustin and Lovinger, 2018). Thus, it is reasonable to speculate that the voltage dependence of the activation of this receptor by 2-AG may play a role in these functions *in vivo*. In this context, we may hypothesize that the agonist specificity of the voltage dependence may underlie the different roles 2-AG and AEA play in some forms of synaptic plasticity. Specifically, it was suggested that AEA is less effective than 2-AG in inducing depolarization induced suppression of excitation (DSE) (Straiker and Mackie, 2009; Luchicchi and Pistis, 2012). Our observation that the potency of 2-AG is higher under depolarization suggests that during depolarization (achieved by the arrival of the action potential to the presynaptic terminal) a low 2-AG concentration is sufficient in order to activate the CB1 receptor and thereby inhibit release. The potency of AEA, on the other hand, remains low even under depolarization and therefore AEA is less effective.

The voltage dependence of the potency of THC may be of pharmacological importance. For example, it has been shown that ultra low levels of THC may have beneficial cognitive effects under some circumstances, for example in mouse were trauma was induced or in models of Alzheimer’s disease (Fishbein et al., 2012; Sarne, 2019; Nitzan et al., 2022). As such conditions also modulate the resting membrane potential, one can speculate that the increased potency of THC under depolarization may play a role in this effect of THC.

## Data Availability

The raw data supporting the conclusion of this article will be made available by the authors, without undue reservation.
