# Supplementary material for: Voltage dependence of the cannabinoid CB1 receptor
Source: Front Pharmacol. 2022 Oct 11;13:1022275. doi: 10.3389/fphar.2022.1022275 (PMC9592857; doi:10.3389/fphar.2022.1022275)
Supplement: Supplementary file 1 [file Table1.DOCX]

**Figure S1.** Effect of 2-AG on GIRK channels. **(A)** A representative recording from an oocyte expressing the GIRK channel. Application of 10 µM 2-AG did not evoke GIRK currents or inhibited basal GIRK currents. **(B)** Current -Voltage (I-V) curve of I_K_ before (black) and after (red) the application of 2-AG. The oocytes were voltage clamped to -80 mV and the currents after depolarizing pulses to various holding potentials from –80 mV to +40 mV at 10 mV increments were measured. Data is mean ± SEM from 7 oocytes. The difference between the two conditions is not significant (paired t-test, *p>*0.11 for all voltages).

**Figure S2.** Effect of AEA on GIRK channels. (**A**) A representative recording from an oocyte expressing the GIRK channel. Application of 10 µM AEA did not evoke GIRK currents or inhibited basal GIRK currents. **(B)** Current-voltage I-V curve of I_K_ before (black) and after (red) the application of AEA. The oocytes were voltage clamped to -80 mV and the currents after depolarizing pulses to various holding potentials from –80 mV to +40 mV at 10 mV increments were measured. Data is mean ± SEM from 7 oocytes. The difference between the two conditions is not significant (paired t-test, *p>*0.13 for all voltages).

**Figure S3.** Effect of THC on GIRK channels. **(A)** A representative recording from an oocyte expressing the GIRK channel. Application of 10 µM THC did not evoke GIRK currents or inhibited basal GIRK currents. **(B)** I-V curve of I_K_ before (black) and after (red) the application of THC. The oocytes were voltage clamped to -80 mV and the currents after depolarizing pulses to various holding potentials from –80 mV to +40 mV at 10 mV increments were measured. Data is mean ± SEM from 5 oocytes. The difference between the two conditions is not significant (paired t-test, *p>*0.23 for all voltages).

**Figure S4.** I-V curve of I_K_ before (empty circles) and after the application of 100 and 10000 nM 2-AG (black and red symbols, respectively), measured from one oocyte. The oocyte was voltage clamped to -80 mV and the currents after depolarizing pulses to various holding potentials from –80 mV to +40 mV at 10 mV increments were measured.
